# Associations of movement behaviors and body mass index: comparison between a report-based and monitor-based method using Compositional Data Analysis

**DOI:** 10.1038/s41366-020-0638-z

**Published:** 2020-07-13

**Authors:** Youngwon Kim, Ryan D. Burns, Duck-chul Lee, Gregory J. Welk

**Affiliations:** 1 School of Public Health, The University of Hong Kong Li Ka Shing Faculty of Medicine, Pokfulam, Hong Kong; 2 MRC Epidemiology Unit, University of Cambridge School of Clinical Medicine, Cambridge, CB2 0QQ Cambridgeshire UK; 3 Department of Health, Kinesiology, and Recreation, University of Utah, Salt Lake City, UT 84112 USA; 4 Department of Kinesiology, Iowa State University, Ames, IA 50011-4008 USA

**Keywords:** Risk factors, Obesity

## Abstract

**Background/objectives:**

Evidence on the associations between lifestyle movement behaviors and obesity has been established without taking into account the time-constrained nature of categorized, time-based lifestyle behaviors. We examined the associations of sleep, sedentary behavior (SED), light-intensity physical activity (LPA), and moderate-to-vigorous PA (MVPA) with body mass index (BMI) using Compositional Data Analysis (CoDA), and compared the associations between a report-based method (24-h Physical Activity Recall; 24PAR) and a monitor-based method (SenseWear Armband; SWA).

**Subjects/methods:**

Replicate data from a representative sample of 1247 adults from the Physical Activity Measurement Survey (PAMS) were used in the study. Participants completed activity monitoring on two randomly selected days, each of which required wearing a SWA for a full day, and then completing a telephone-administered 24PAR the following day. Relationships among behavioral compositional parts and BMI were analyzed using CoDA via multiple linear regression models with both 24PAR and SWA data.

**Results:**

Using 24PAR, time spent in sleep (*γ* = −3.58, *p* = 0.011), SED (*γ* = 3.70, *p* = 0.002), and MVPA (*γ* = −0.53, *p* = 0.018) was associated with BMI. Using SWA, time spent in sleep (*γ* = −5.10, *p* < 0.001), SED (*γ* = 8.93, *p* < 0.001), LPA (*γ* = −3.12, *p* < 0.001), and MVPA (*γ* = −1.43, *p* < 0.001) was associated with BMI. The SWA models explained more variance in BMI (*R*^2^ = 0.28) compared with the 24PAR models (*R*^2^ = 0.07). The compositional isotemporal substitution models revealed reductions in BMI when replacing SED by MVPA, LPA (not with 24PAR) or sleep for both 24PAR and SWA, but the effect estimates were larger with SWA.

**Conclusions:**

Favorable levels of relative time spent in lifestyle movement behaviors were, in general, associated with decreased BMI. The observed associations were stronger using the monitor-based SWA method compared with the report-based 24PAR method.

## Introduction

Nearly one in every three US adults is considered obese, and the prevalence is expected to increase over the next two decades [1]. Numerous factors have contributed to the obesity epidemic, but a crucial determinant is the rapid societal and environmental changes from physically active lifestyles to sedentary lifestyles. This transition has been attributable, in large part, to the drastic changes in technology, the increased reliance on motor vehicles, and the reduced activity involved in contemporary office work [2, 3].

Estimates suggest that US adults spend almost 8 h a day on sedentary behavior (SED) which accounts for ~55% of waking hours per day [4]. Evidence indicates that excess time spent sedentary has detrimental effects on cancer, cardiovascular disease, and mortality risk in adults, irrespective of the accumulation of moderate-to-vigorous physical activity (MVPA) [5]. In addition, studies have demonstrated that obese individuals tend to spend more time being sedentary and less time being physically active than normal weight or overweight individuals [6, 7]. These findings hint at the complex etiology underlying the health impacts of SED on obesity. Research on SED is still in a relatively early phase of development and is plagued by measurement challenges and lack of consensus on operational definitions [8].

To date, typical studies have only investigated SED and MVPA as salient time-use compositional behaviors relative to weight-related outcomes. However, there are other salient time-use compositional behaviors such as sleeping (SLEEP) [9] and light physical activity (LPA) [10] that have been recently recognized as predictors of cardiometabolic risk markers. However, there is a paucity of work examining the concurrent and compositional relationships among SLEEP, SED, LPA, and MVPA on weight-related outcomes, particularly in a representative sample of adult populations.

Most large-scale epidemiological studies on obesity have used self-report methods, which are known to be more prone to measurement errors than accelerometry-based activity monitors. Differences in activity outcomes from report-based and monitor-based measures of PA have been well-documented. For instance, the proportion of US adults meeting the PA guidelines has been estimated at over 50% with self-report data, but is <5% when assessed with accelerometry [11]. A few studies have evaluated associations between SED and obesity with both report-based and monitor-based measures, but findings have been inconsistent [12–14]. The use of count-based methods for processing accelerometer data may contribute to some of the equivocal findings since this approach has some limitations for quantifying SED [15]. The use of long-term recall measures in these studies [12–14] may also contribute to equivocal findings, since these methods are associated with greater measurement errors than short-term recall methods [16]. The types of measures being compared and the sophistication of the data processing methods can dramatically influence activity and health outcomes, so additional research is needed to better understand the relationships between lifestyle behaviors and weight-related outcomes. The use of Compositional Data Analysis (CoDA) offers advantages for evaluating these relationships since it can account for the compositional properties of time-based behaviors [17–20]. More specific to obesity research, CoDA enables the impact of differences in time allocation on weight status to be empirically evaluated.

To fill the gaps in this field of research, it is critical to understand the extent to which the choice of measurement tools affects the interplay of time-use compositional behaviors of SLEEP, LPA, SED, and MVPA in relation to BMI at the population level. Therefore, the purposes of this study were (1) to determine the relationships between movement-related time-use components and BMI using CoDA, and (2) to examine whether the observed CoDA associations vary by type of assessment methods in a representative sample of adults.

### Materials/subjects and methods

This research is an ancillary study of the Physical Activity Measurement Survey (PAMS) project, a cross-sectional survey study that has examined relationships between monitor-based and report-based measures of PA [21, 22] and SED [23, 24]. The data collection for the PAMS project was carried out across eight consecutive, 3-month quarters (i.e., 2 years) to capture seasonal/weather variations. Participants completed replicate trials consisting of 24-h activity monitor use, followed by a 24-h recall survey on two randomly selected days within one of the quarters of data collection. The PAMS project was approved by the local Institutional Review Board and is described in greater detail elsewhere [21–24]. Each participant provided signed written informed consent prior to participation.

### Participants

The PAMS project employed a multi-level stratified sampling technique to recruit a representative sample of adults across four counties (two rural and two urban) in Iowa, USA. Adults included in a purchased sample pool from Survey Sampling International were contacted via random digit dialing. The inclusion criteria were adults aged between 20 and 75 years, and capable of walking and completing surveys either in English or Spanish. The exclusion criteria were adults with any critical medical conditions preventing them from engaging in PA. The sampling and characteristics have been previously described, but the present study necessitated the use of replicate samples so the final sample included 1247 participants (See Supplementary Fig. 1).

### Instruments

Two established measurement tools were used to obtain both monitor-based and report-based measures of movement behaviors.

#### SenseWear Armband Mini (SWA)

The SWA (BodyMedia, Inc., Pittsburgh, PA) is a non-invasive pattern-recognition monitoring tool that utilizes multiple sensors (heat flux, galvanic skin response, skin temperature, near body temperature) as well as a tri-axial accelerometer. The SWA provides a variety of activity parameters (i.e., activity time, MET, Kcal, speed, and distance) for every minute. The accuracy of the SWA for adults has been tested in previous studies [25–27]. Data from the SWA were processed using the latest version of Software v8.0 (coupled with the proprietary algorithms v5.2).

#### 24 physical activity recall (24PAR)

The 24PAR is a self-report tool designed to assess activity time, energy expenditure, and context of activities performed in the previous day. The 24PAR was administered over the telephone by trained interviewers using a computer-assisted telephone interview system programmed with the *Blaise* software. The 24PAR interview requires each participant to report on the past day’s activities in episodes of at least 5 minutes. The accuracy of 24PAR has been tested for assessing activity time and energy expenditure [25, 28, 29].

### Data collection

The PAMS project used a 24-h monitoring protocol to directly link data from the SWA to the data from the 24PAR. Participants wore the SWA for 24-h on a randomly selected day, and completed the 24PAR assessment the following day to recall and report on activities performed in the previous day (i.e., the same day as the SWA monitoring day). Field staff members were sent to each participant’s home (the day before the SWA monitoring day) to provide detailed descriptions of the PAMS protocol, and distribute a SWA monitor. The staff also provided each participant an activity log to record any activities performed while the SWA was not worn. A follow-up visit to the participant’s home occurred the day after the monitoring day to collect the SWA monitor used. Participants completed a second trial on another randomly selected day (at least 12 days after the first measurement trial) to obtain replicate measures from both the SWA and 24PAR.

### Data processing

Upon completion of the SWA monitoring protocol, the SWA data were downloaded using the proprietary algorithms/software. The SWA provides MET values for every minute, so the standard MET-derived criteria were applied to classify each minute into different intensity categories: ≤1.5MET for SED, 1.5 < MET < 3.0 for LPA, and ≥3.0MET for MVPA. Classified minutes were aggregated to obtain total daily activity minutes of the respective intensities. SWA-determined sleep time (SLEEP) was subtracted from the total categorized sedentary time to produce SED for the SWA.

The PAMS protocol used a reduced listing of 270 codes from the Compendium of PA [21, 30] to assign predicted MET values to each reported activity from the 24PAR interview. The same standard MET criteria were used for the 24PAR data to classify each reported activity into the different intensities. The corresponding estimate of SED from the 24PAR was obtained by subtracting self-reported SLEEP time from the sum of all minutes reported for the 27 specific sedentary activities (i.e., ≤1.5MET). LPA time for 24PAR was estimated by subtracting SLEEP, SED, and MVPA time from 1440 minutes. To obtain more stable estimates of each movement behavior, the average values from the two independent days of testing were computed for each participant. Body Mass Index (BMI) was calculated as measured weight (kg)/height squared (m^2^). Individuals with BMI ≥ 30 and BMI < 30 were classified as obese and non-obese, respectively. Key covariates controlled for in the statistical models included gender (i.e., male and female), age (years), ethnicity (i.e., White, Black, and Other), annual income (i.e., <$25,000/year, between $25,000 and $75,000/year, >$75,000/year), employment (i.e., full-time, part-time, and unemployed/retired/full-time homemaker), education background (i.e., <high school, some college/post-high school, and college/graduate), marital status (i.e., married/living as married, divorced/separated/widowed, and single/never married), current smoking status (i.e., smoker and non-smoker), and the measurement day of the week (i.e., 2 weekdays, 2 weekend days, and 1 weekday and 1 weekend day).

### Methods for Compositional Data Analyses (CoDA)

The following CoDA procedures have been adopted from prior published work [19, 31–33]. Minutes per day in SLEEP, SED, LPA, and MVPA were converted to % of wear-time in each respective compositional part so that the sum was equal to 100%. The geometric mean (in min/day) was calculated for all parts and the sum of each part was adjusted to 1440 minutes. Therefore, the compositional value represents % time-use out of a 24-h day. Compositional data occupy a quotient space which can be represented in a D-part simplex with four compositional parts (4-part simplex) [19, 31]. However, in order to analyze the data in real space, log-ratio data transformations needed to be performed. Isometric Log Ratio coordinates (ILRs) were calculated using the following equations:

1 $${\mathrm{ILR}}1 = \sqrt {\frac{3}{4}} \;{\mathrm{ln}}\left( {\frac{{{\mathrm{SLEEP}}}}{{\left( {{\mathrm{SED}} \times {\mathrm{LPA}} \times \,{\mathrm{MVPA}}} \right)^{\frac{1}{3}}}}} \right),$$

2 $${\mathrm{ILR}}2 = \sqrt {\frac{2}{3}}\; {\mathrm{ln}}\left( {\frac{{{\mathrm{SED}}}}{{\left( {{\mathrm{LPA}} \times {\mathrm{MVPA}}} \right)^{\frac{1}{2}}}}} \right),$$

3 $${\mathrm{ILR}}3 = \sqrt {\frac{1}{2}}\; {\mathrm{ln}}\left( {\frac{{{\mathrm{LPA}}}}{{{\mathrm{MVPA}}}}} \right).$$

ILR1 expresses time in SLEEP to time in all other non-SLEEP behaviors. ILR2 is the ratio of SED in relation to LPA and MVPA. Finally, ILR3 is the ratio of LPA to MVPA. These 3 ILRs were included in the linear regression models described below to obtain the corresponding parameter estimates. However, the inference about the primary contrast of interest in this set of analyses (i.e., SLEEP relative to the three non-SLEEP behaviors) was based purely on ILR1. As such, additional ILRs were calculated through a parallel set of equations by permutating the compositional parts in a sequential manner to obtain parameter estimates for the other three major behaviors of interest: SED (ILRs 4–6), LPA (ILRs 7–9) and MVPA (ILRs 10–12) [17].

4 $${\mathrm{ILR}}4 = \sqrt {\frac{3}{4}}\; {\mathrm{ln}}\left( {\frac{{{\mathrm{SED}}}}{{\left( {{\mathrm{SLEEP}} \times {\mathrm{LPA}} \times {\mathrm{MVPA}}} \right)^{\frac{1}{3}}}}} \right),$$

5 $${\mathrm{ILR}}5 = \sqrt {\frac{2}{3}}\; {\mathrm{ln}}\left( {\frac{{{\mathrm{SLEEP}}}}{{\left( {{\mathrm{LPA}} \times {\mathrm{MVPA}}} \right)^{\frac{1}{2}}}}} \right),$$

6 $${\mathrm{ILR}}6 = \sqrt {\frac{1}{2}}\;{\mathrm{ln}}\left( {\frac{{{\mathrm{LPA}}}}{{{\mathrm{MVPA}}}}} \right),$$

7 $${\mathrm{ILR}}7 = \sqrt {\frac{3}{4}}\; {\mathrm{ln}}\left( {\frac{{{\mathrm{LPA}}}}{{\left( {{\mathrm{SLEEP}} \times {\mathrm{SED}} \times {\mathrm{MVPA}}} \right)^{\frac{1}{3}}}}} \right),$$

8 $${\mathrm{ILR}}8 = \sqrt {\frac{2}{3}}\; {\mathrm{ln}}\left( {\frac{{{\mathrm{SLEEP}}}}{{\left( {{\mathrm{SED}} \times {\mathrm{MVPA}}} \right)^{\frac{1}{2}}}}} \right),$$

9 $${\mathrm{ILR}}9 = \sqrt {\frac{1}{2}}\; {\mathrm{ln}}\left( {\frac{{{\mathrm{SED}}}}{{{\mathrm{MVPA}}}}} \right),$$

10 $${\mathrm{ILR}}10 = \sqrt {\frac{3}{4}}\; {\mathrm{ln}}\left( {\frac{{{\mathrm{MVPA}}}}{{\left( {{\mathrm{SLEEP}} \times {\mathrm{SED}} \times {\mathrm{LPA}}} \right)^{\frac{1}{3}}}}} \right),$$

11 $${\mathrm{ILR}}11 = \sqrt {\frac{2}{3}}\; {\mathrm{ln}}\left( {\frac{{{\mathrm{SLEEP}}}}{{\left( {{\mathrm{SED}} \times {\mathrm{LPA}}} \right)^{\frac{1}{2}}}}} \right),$$

12 $${\mathrm{ILR}}12 = \sqrt {\frac{1}{2}}\; {\mathrm{ln}}\left( {\frac{{{\mathrm{SED}}}}{{{\mathrm{LPA}}}}} \right).$$

Therefore, the ILRs from Eqs. (1–3) were entered into each linear model to obtain the parameter estimate for ilr _SLEEP_, the ILRs from Eqs. (4–6) were entered into each linear model to obtain the parameter estimate for ilr _SED_, the ILRs from Eqs. (7–9) were entered into each linear model to obtain the parameter estimate for ilr _LPA_, and the ILRs from Eqs. (10–12) were entered into each linear model to obtain the parameter estimate for ilr _MVPA_. Because of the permutation principle, each respective linear model with 4 compositional parts (SLEEP, SED, LPA, MVPA) will have the same estimated fit, intercept, and *p* value for all covariates per permutation [17]. This process was carried out using the modeling procedures described below for both 24PAR and SWA.

### Statistical analyses

Arithmetic and geometric means for the four behavior compositional parts were reported. Compositional mean bar plots were created to display the log ratio of geometric means, per obesity strata (non-obese, obese), to the mean of the whole sample [17, 32, 33]. Bar plots were derived for both 24PAR and SWA. Compositional variation matrices were used to communicate the variation of the calculated pair-wise log ratios (e.g., ln (SED/MVPA)) [17, 32, 33]. A variation coefficient closer to 0 would indicate that there is higher co-dependency between two respective compositional parts. Higher co-dependency suggests that there is smaller variability in the log ratio between the two compositional parts within the sample [17, 32, 33]. Total variance within the matrices was calculated by dividing the sum of the variances on either side of the diagonal by *D* = 4 [19, 31].

To examine the relationship among SLEEP, SED, LPA, MVPA, and BMI, multiple linear regression models were employed. Separate models were employed for 24PAR and SWA. Four permutations were used to calculate parameter estimates for each of the four compositional parts. Parameter estimates (gamma coefficients) and corresponding 95% confidence intervals were reported. Models were adjusted for age, sex, ethnicity, income, employment, education, marital status, smoking status, and the measurement day of the week. Model fit was examined using the coefficient of determination (*R*^2^). A series of logistic regression models combined with the same sequential permutation procedure was performed to examine the relationships of the compositional parts with a binary BMI-determined obesity status outcome (defined using the 30 kg/m^2^ cut point).

Because parameter estimates for ILRs are difficult to interpret in the context of units of change in the raw behaviors [17, 33], compositional isotemporal substitution, as recommended and outlined by Dumuid et al. [20], was used to determine how reallocation of time spent between behaviors is associated with changes in BMI. Means for each compositional part were used to predict BMI values from which BMI change values could be calculated based on a new composition (e.g., reallocating 10 min from SED to MVPA). Data using compositional isotemporal substitution were used to derive plots indicating how reallocation of time in the ratio between any two compositional parts, with a fixed relative amount of the third and fourth compositional part, is associated with a predicted change in BMI (via the adjusted linear model) and the odds of obesity (via the adjusted logistic model). Technical details of this specific CoDA calculation have been described elsewhere [32]. All analyses had a statistical significance level set at *p* < 0.05 and were carried out using STATA v15.0 statistical software package (StataCorp LLC, College Station, TX).

## Results

Table 1 summarizes the physical/socio-demographic characteristics of the non-obese and obese individuals. About 44.4% of the participants were obese. There were significant differences between non-obese and obese groups on income, education, and smoking status (*p* < 0.05). There were no significant differences between non-obese and obese groups on gender, age, ethnicity, employment status, marital status, and measurement day of the week.

**Table 1:** Characteristics of the participants for all and by weight status: non-obesity (*n* = 694) and obesity (*n* = 553).

	All	Non-obesity	Obesity	*p* value
Gender, *n* (%)				
Female	714 (57.3)	398 (57.4)	316 (57.1)	0.942
Male	533 (42.7)	296 (42.6)	237 (42.9)	
Age, years	50.1 (12.5)	49.5 (13.0)	50.8 (11.8)	0.0686
BMI, kg/m^2^	30.4 (7.4)	25.5 (3.0)	36.7 (6.4)	
Ethnicity, *n* (%)				
White	1,116 (89.5)	626 (90.2)	490 (88.6)	0.156
Black	95 (7.6)	45 (6.5)	50 (9.0)	
Other	36 (2.9)	23 (3.3)	13 (2.4)	
Income, *n* (%)				
<$25,000/yr	223 (17.9)	115 (16.6)	108 (19.5)	0.023^a^
From $25,000 up to $75,000/yr	606 (48.6)	324 (46.7)	282 (51.0)	
>$75,000/yr	418 (33.5)	255 (36.7)	163 (29.5)	
Employment, *n* (%)				
Full time	741 (59.4)	401 (57.8)	340 (61.5)	0.142
Part time	168 (13.5)	105 (15.1)	63 (11.4)	
Unemployed/retired/full time homemaker	338 (27.1)	188 (27.1)	150 (27.1)	
Education, *n* (%)				
Less than high school	51 (4.1)	25 (3.6)	26 (4.7)	<0.001^a^
High school diploma/some college	708 (56.8)	363 (52.3)	345 (62.4)	
College/graduate school	488 (39.1)	306 (44.1)	182 (32.9)	
Marital status, *n* (%)				
Married/living as married	820 (65.7)	460 (66.3)	360 (65.1)	0.787
Divorced/separated/widowed	224 (18.0)	120 (17.3)	104 (18.8)	
Single/never married	203 (16.3)	114 (16.4)	89 (16.1)	
Current Smoking status, *n* (%)				
Yes	235 (18.9)	151 (21.8)	84 (15.2)	0.003^a^
No	1,012 (81.1)	543 (78.2)	469 (84.2)	
Measurement Day of week, *n* (%)				
2 Weekdays	702 (56.3)	389 (56.1)	313 (56.6)	0.558
2 Weekend days	97 (7.8)	59 (8.5)	38 (6.9)	
1 Weekday + 1 Weekend day	448 (35.9)	246 (35.4)	202 (36.5)	

*24PAR* 24-h physical activity recall, *SWA* *SenseWear Armband*, *SLEEP* time sleeping, *SED* sedentary time, *LPA* light physical activity, *MVPA* moderate-to-vigorous physical activity.

^a^Indicates significant relationships based on the Chi-square tests (an alpha level = 5%).

**Fig. 1:**
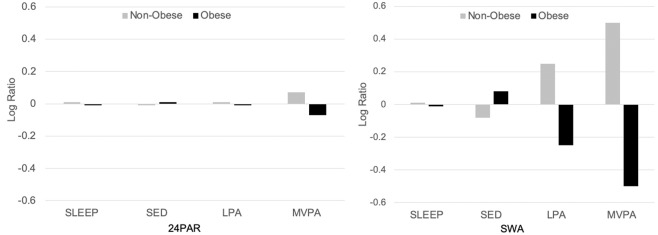
Geometric mean barplots (24PAR-Left, SWA-Right) showing time spent sleeping, in sedentary behavior, in light physical activity, and in moderate-to-vigorous physical activity, stratified by obesity status. Each bar represents the geometric mean of the specific group (*g*_k_), expressed in terms of a ratio measured on a logarithmic scale to the geometric mean of the entire sample for each behavior (ln *g*_k_/*g*). A ratio of 0 reflects that the geometric means of the specific group and the entire sample are equal. Positive and negative values show that the group geometric mean is larger and smaller, respectively, than the entire sample, 24PAR stands for 24-h Physical Activity Recall, SLEEP is time sleeping, SED stands for Sedentary Time, LPA stands for Light Physical Activity, MVPA stands for Moderate-to-Vigorous Physical Activity.

Table 2 communicates the Arithmetic and Geometric means for SLEEP, SED, LPA, and MVPA for 24PAR and SWA. The majority of the day was spent in SED, followed by SLEEP, LPA, and MVPA. Table 3 communicates the compositional variation matrices using 24PAR and SWA. Coefficients within the variation matrices ranged from 0.21 to 1.56. In general, coefficients were higher for the SWA compared with the 24PAR, indicting greater independence between compositional parts. This is reflected in the higher total variation observed using SWA (1.51) compared with 24PAR (1.23). The highest coefficient was the log ratio variance between SED and MVPA using SWA (indicating high independence between parts) and the smallest was the log ratio variance between SED and SLEEP using 24PAR (indicating high co-dependence between parts). Descriptive differences comparing Geometric means between non-obese and obese individuals using 24PAR (i.e., left panel) and SWA (i.e., right panel) are presented in Fig. 1. Obese individuals spent more time in SED and less time in LPA and MVPA. Group contrasts for SLEEP were negligible. Greater contrasts between non-obese and obese groups were observed using SWA.

**Table 2:** Arithmetic and geometric compositional means for the four compositional parts (in minutes/day and % of total day).

	Arithmetic mean		Compositional mean	
	Minutes/day	%	Minutes/day	%
24-h Physical Activity Recall (24PAR)				
SLEEP	361.4	25.1%	377.3	26.2%
SED	705.6	49.0%	733.0	50.9%
LPA	266.4	18.5%	253.4	17.6%
MVPA	106.6	7.4%	76.3	5.3%
SenseWear Armband (SWA)				
SLEEP	296.6	20.6%	299.5	20.8%
SED	819.4	56.9%	874.1	60.7%
LPA	246.2	17.1%	224.6	15.6%
MVPA	77.7	5.4%	41.8	2.9%

*SLEEP* time sleeping; *SED* sedentary time; *LPA* light physical activity; *MVPA* moderate-to-vigorous physical activity.

**Table 3:** Compositional variation matrices.

	SLEEP	SED	LPA	MVPA
24-h Physical Activity Recall (24PAR)				
SLEEP	0			
SED	0.21	0		
LPA	0.57	0.65	0	
MVPA	1.10	1.17	1.22	0
SenseWear Armband (SWA)				
SLEEP	0			
SED	0.30	0		
LPA	0.75	0.78	0	
MVPA	1.49	1.56	1.17	0

Lower cell values indicate greater co-dependence between compositional parts; higher cell values indicate greater independence between compositional parts.

Total variance: 24PAR = 1.23, SWA = 1.51.

*SLEEP* time sleeping, *SED* sedentary time, *LPA* light physical activity, *MVPA* moderate-to-vigorous physical activity.

Table 4 shows the unadjusted and adjusted parameter estimates (gamma-coefficients and 95% confidence intervals) from the linear regression models performed using the CoDA approach. Using 24PAR, after adjusting for potential confounding variables, time spent in SLEEP (*p* = 0.011), SED (*p* = 0.002), and MVPA (*p* = 0.018) were all significantly associated with BMI. Specifically, individuals who had a higher log ratio of SLEEP or MVPA over other compositional parts tended to have lower BMI. Conversely, individuals who had a higher log ratio of SED over other compositional parts tended to have higher BMI. The adjusted 24PAR model *R*^2^ was 0.075. Using SWA, all ILRs in the adjusted model were statistically significant predictors of BMI; the corresponding coefficients were larger compared with those using 24PAR. Specifically, individuals who had a higher log ratio of SLEEP, LPA, or MVPA over other compositional parts tended to have lower BMI (*p* < 0.001). Individuals who had a higher ratio of SED over other compositional parts tended to have higher BMI (*p* < 0.001). The adjusted SWA model *R*^2^ was 0.283.

**Table 4:** Parameter estimates from the Body Mass Index multiple linear regression models using compositional data analyses (reported as gamma-coefficients).

Assessment Method	Isometric Log Ratio Predictor	Unadjusted Model *γ*—coefficient (95% CI)	Adjusted Model *γ*—coefficient (95% CI)	*p* value
24-h Physical Activity Recall (24PAR)	ilr _SLEEP/SED*LPA*MVPA_	**−3.38**^**a**^ **(−6.14, −0.63)**	**−3.58**^**a**^ **(−6.29, −0.79)**	0.011
	ilr _SED/SLEEP*LPA*MVPA_	**3.58**^**a**^ **(1.26, 5.91)**	**3.70**^**a**^ **(1.37, 5.99)**	0.002
	ilr _LPA/SLEEP*SED*MVPA_	0.27 (−0.61, 1,14)	0.41 (−0.51, 1.31)	0.541
	ilr _MVPA/SLEEP*SED*LPA_	**−0.47**^**a**^ **(−0.91, −0.03)**	**−0.53**^**a**^ **(−0.99, −0.09)**	0.018
SenseWear Armband (SWA)	ilr _SLEEP/SED*LPA*MVPA_	**−5.62**^**a**^ **(−6.95, −4.30)**	**−5.10**^**a**^ **(−6.40, −3.84)**	<0.001
	ilr _SED/SLEEP*LPA*MVPA_	**9.24**^**a**^ **(7.83, 10.66)**	**8.93**^**a**^ **(7.57, 10.31)**	<0.001
	ilr _LPA/SLEEP*SED*MVPA_	**−3.36**^**a**^ **(−4.11, −2.62)**	**−3.12**^**a**^ **(−3.85, −2.40)**	<0.001
	ilr _MVPA/SLEEP*SED*LPA_	**−0.74**^**a**^ **(−1.08, −0.38)**	**−1.43**^**a**^ **(−1.80, −1.06)**	<0.001

Models were adjusted for age, sex, ethnicity, income, employment, education, marital status, smoking status, and measurement day of the week.

Outcome is Body Mass Index (kg/m^2^).

*SLEEP* time sleeping, *SED* sedentary time, *LPA* light physical activity, *MVPA* moderate-to-vigorous physical activity, *ilr* isometric log ratio, *95% CI* 95% confidence interval.

^a^Bold and denotes statistical significance (an alpha level = 5%).

**Fig. 2:**
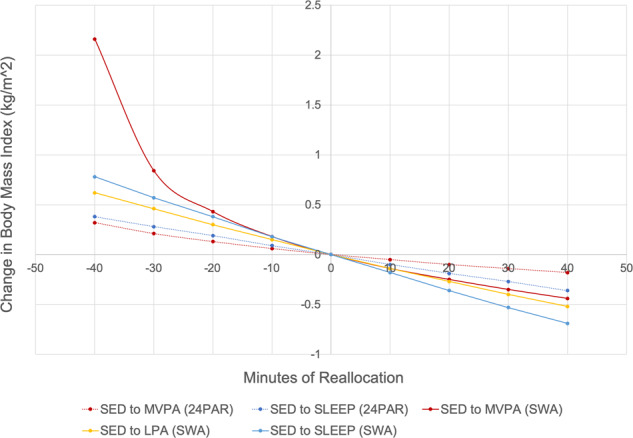
Change in Body Mass Index (kg/m^2^) for every 10-min reallocation of time-use compositional behavior using 24-h Physical Activity Recall and SenseWear Armband. SLEEP is time sleeping; SED stands for Sedentary Time; LPA stands for Light Physical Activity; MVPA stands for Moderate-to-Vigorous Physical Activity. Note: Dashed lines are 24PAR; solid lines are SWA; LPA not shown for 24PAR due to non-significance.

Figure 2 was derived using the compositional isotemporal substitution method outlined in Dumuid et al. [20] indicating how reallocating time from SED to SLEEP, LPA or MVPA is associated with a change in BMI for data from both the 24PAR and SWA. Specific BMI change values for 10- and 30-min reallocation of time are presented in Table 5 using values reported in Fig. 2. Using 24PAR, a 10-min reallocation from SED to MVPA and from SED to SLEEP was associated with 0.05- and 0.10-unit lower BMI, respectively. Using SWA, a 10-min reallocation from SED to MVPA or LPA was associated with 0.14-unit lower BMI; and 0.18-unit lower BMI for a 10-min reallocation from SED to SLEEP (Table 5). The effect estimates were larger, indicating stronger associations, when using data from SWA compared with 24PAR. An identical trend of associations was observed for 30-min reallocation (Table 5 and Fig. 2). Results from the logistic regression models using BMI as a categorical variable (e.g., obesity versus non-obesity) also identified stronger associations when reallocating time between behaviors using SWA than 24PAR (See Supplementary Fig. 2 and Supplementary Table 1).

**Table 5:** Predicted changes in Body Mass Index (BMI) (kg/m^2^) following 10-min and 30-min reallocation between behaviors using compositional isotemporal substitution.

	10-min reallocation	30-min reallocation
24-h Physical Activity Recall (24PAR)		
SED to MVPA	−0.05	−0.14
MVPA to SED	0.06	0.21
SED to SLEEP	−0.10	−0.27
SLEEP to SED	0.09	0.28
SenseWear Armband (SWA)		
SED to MVPA	−0.14	−0.35
MVPA to SED	0.18	0.84
SED to LPA	−0.14	−0.40
LPA to SED	0.15	0.46
SED to SLEEP	−0.18	−0.53
SLEEP to SED	0.18	0.57

24PAR LPA not shown because of no statistical significance.

*SLEEP* time sleeping, *SED* sedentary time, *LPA* light physical activity, *MVPA* moderate-to-vigorous physical activity.

## Discussion

This study is the first empirical investigation to use the CoDA procedures to examine the associations of lifestyle behaviors as compositional parts with weight status using both a report- and monitor-based assessment method. When time-use compositional behaviors were assessed using the 24PAR, only relative time spent in SLEEP, SED, and MVPA (not LPA) had significant associations with BMI. The compositional isotemporal substitution modeling suggests that replacing SED by either SLEEP or MVPA is associated with lower BMI. These findings add much value to previous research which examined obesity in relation to self-reported MVPA or SED as individual, non-dependent behavioral constructs using traditional regression models [14, 34–36]. Another unique advantage of this study is the ability to compare outcomes from both report-based and monitor-based data with CoDA.

The monitor-based estimates of SLEEP, SED, and MVPA from the SWA were found to be more strongly associated with BMI, compared with 24PAR. The weaker associations with 24PAR may be indicative of the larger measurement error associated with 24PAR due to the recall bias [37] and social desirability and approval bias [38]. In support, large error in exposure variables (e.g., behaviors) is known to lead to reductions in effect estimates [39]. In addition, the compositional isotemporal substitution modeling with SWA demonstrated reduced BMI when SED was replaced not only by SLEEP or MVPA time but also by LPA time. These results suggest that reductions in obesity can be achieved by substituting sedentary time with light-intensity lifestyle activities (i.e., standing, slow walking). Promoting changes in MVPA time is relatively more difficult, since it requires adoption of more purposive, higher intensity activities (e.g., sports, brisk walking, or running). From a public health perspective, encouraging shifts from SED to LPA may be a more efficient and practical prevention strategy than promoting shifts from SED to MVPA to attenuate the obesity epidemic. However, replacing SED with both LPA and MVPA may have a more significant impact on energy expenditure than just replacing SED with LPA alone.

The direct contrast between 24PAR and SWA herein adds to findings from past research [12–14] which attempted to examine the differences in associations between MVPA or SED and metabolic risk factors according to the use of different PA assessment methods. However, none of them [12–14] used the CoDA to address the time-constraint nature of lifestyle behaviors. Moreover, they [12–14] used vastly different methodologies (i.e., types of self-report tools, sedentary activities, accelerometry count cut-offs, populations), which makes it challenging to make a fair comparison across all investigations. However, a distinguishable feature of the present research is the use of pattern-recognition monitors and the 24PAR, both of which are known to be more accurate than traditional accelerometers [40] and long-term recalls [21], respectively. Further investigations are needed to better understand the implications of utilizing different measurement tools in identifying associations among lifestyle behaviors and BMI using CoDA.

The strengths of the study include the large, representative nature of the sample, the use of CoDA to examine the relationships between time-use compositional behaviors and BMI, and robust analyses controlling for several potential confounding variables. However, there are some limitations of this study that should be considered when interpreting the results. First, given that this study capitalized on cross-sectional data, no causal inferences can be drawn. The directions of causal relationships among SLEEP, SED, LPA, MVPA, and BMI can still not be determined [41, 42]. There has been only few research [43] using the CoDA to investigate the longitudinal associations of objectively measured time-use compositional behaviors with adiposity, so more prospective cohort studies incorporating objective, repetitive measures of movement behaviors are needed to determine the true relationships of constrained time-use compositional behaviors relative to various adiposity indicators [44]. Another limitation is the assessment across only two days, which may not capture the routine activity levels of the participants. However, the two measurement days were randomly selected, and the data were collected over a 2-year time span to adjust for the potential weather/seasonal variation in activity patterns. Nevertheless, there could still be day-to-day variability for each participant that incorrectly characterizes individual profiles. These errors would tend to be random across the sample so results would likely be stronger if more days were assessed. The majority of the participants were Caucasian (89%), and individuals in the present study were relatively physically active (i.e., about 1 h/day MVPA in non-obese and 2 h/day of MVPA in obese individuals), so it may be premature to assume that these relationships hold in other populations. Another limitation is that the results are only capturing the associations of lifestyle activities with BMI. The lack of information about dietary intake limits our ability to fully understand energy balance and weight control.

## Conclusions

The compositional associations among the time-use compositional behaviors of SLEEP, SED, MVPA, with BMI are much stronger using SWA compared to 24PAR. BMI had strong associations with relative time-use within monitor-based estimates of LPA, but not with self-reported LPA. Replacing SED by SLEEP, LPA (not with 24PAR), or MVPA was associated with reduced BMI, but the effect estimates were much weaker when using 24PAR compared with SWA. This may be attributable to the substantial measurement errors inherent in 24PAR. Error in report-based measures of constrained time-use compositional behaviors may obscure the clinically important obesity associations.

## Supplementary information

Supplement
