# Supplementary material for: Associations of movement behaviors and body mass index: comparison between a report-based and monitor-based method using Compositional Data Analysis
Source: Int J Obes (Lond). 2020 Jul 13;45(1):266–75. doi: 10.1038/s41366-020-0638-z (PMC7752757; doi:10.1038/s41366-020-0638-z)
Supplement: Supplementary file 1 — Supplement [file 41366_2020_638_MOESM1_ESM.docx]

**Supplementary Table 1.** Parameter estimates from the logistic regression models using a binary compositional data analysis.

| Assessment | Isometric Log Ratio Predictor | Unadjusted Model  Odds Ratio (OR)  (95% CI) | Adjusted Model  Odds Ratio (OR)  (95% CI) | *p*-value |
| --- | --- | --- | --- | --- |
| 24PAR | ilr _SLEEP_ | 0.47 (0.21, 1.03) | 0.49 (0.21, 1.09) | 0.068 |
|  | ilr _SED_ | **1.97^†^ (1.02, 3.82)** | 1.90 (0.98, 3.86) | 0.064 |
|  | ilr _LPA_ | 1.18 (0.92, 1.52) | 1.23 (0.94, 1.62) | 0.141 |
|  | ilr _MVPA_ | 0.91 (0.81, 1.03) | **0.86^†^ (0.76, 0.99)** | 0.032 |
| SWA | ilr _SLEEP_ | **0.14^†^ (0.08, 0.24)** | **0.14^†^ (0.08, 0.26)** | < 0.001 |
|  | ilr _SED_ | **18.83^†^ (10.24, 34.64)** | **22.6^†^ (11.78, 42.84)** | < 0.001 |
|  | ilr _LPA_ | **0.38^†^ (0.28, 0.52)** | **0.38^†^ (0.28, 0.53)** | < 0.001 |
|  | ilr _MVPA_ | **0.83^†^ (0.72, 0.96)** | **0.63^†^ (0.54, 0.76)** | < 0.001 |

Abbreviations: 24PAR - 24-hour Physical Activity Recall; SWA - SenseWear Armband; SLEEP - time sleeping; SED - sedentary times; LPA - Light Physical Activity; MVPA - Moderate-to-Vigorous Physical Activity; ilr – Isometric Log Ratio; 95% CI – 95% Confidence Interval.

Adjusted Model accounts for age, sex, ethnicity, income, employment, education, marital status, smoking status, and measurement day of the week.

Outcome is Body Mass Index determined obesity status (cut-point < 30 kg/m^2^).

Bold and ^†^ denotes statistical significance (an alpha level = 5%).


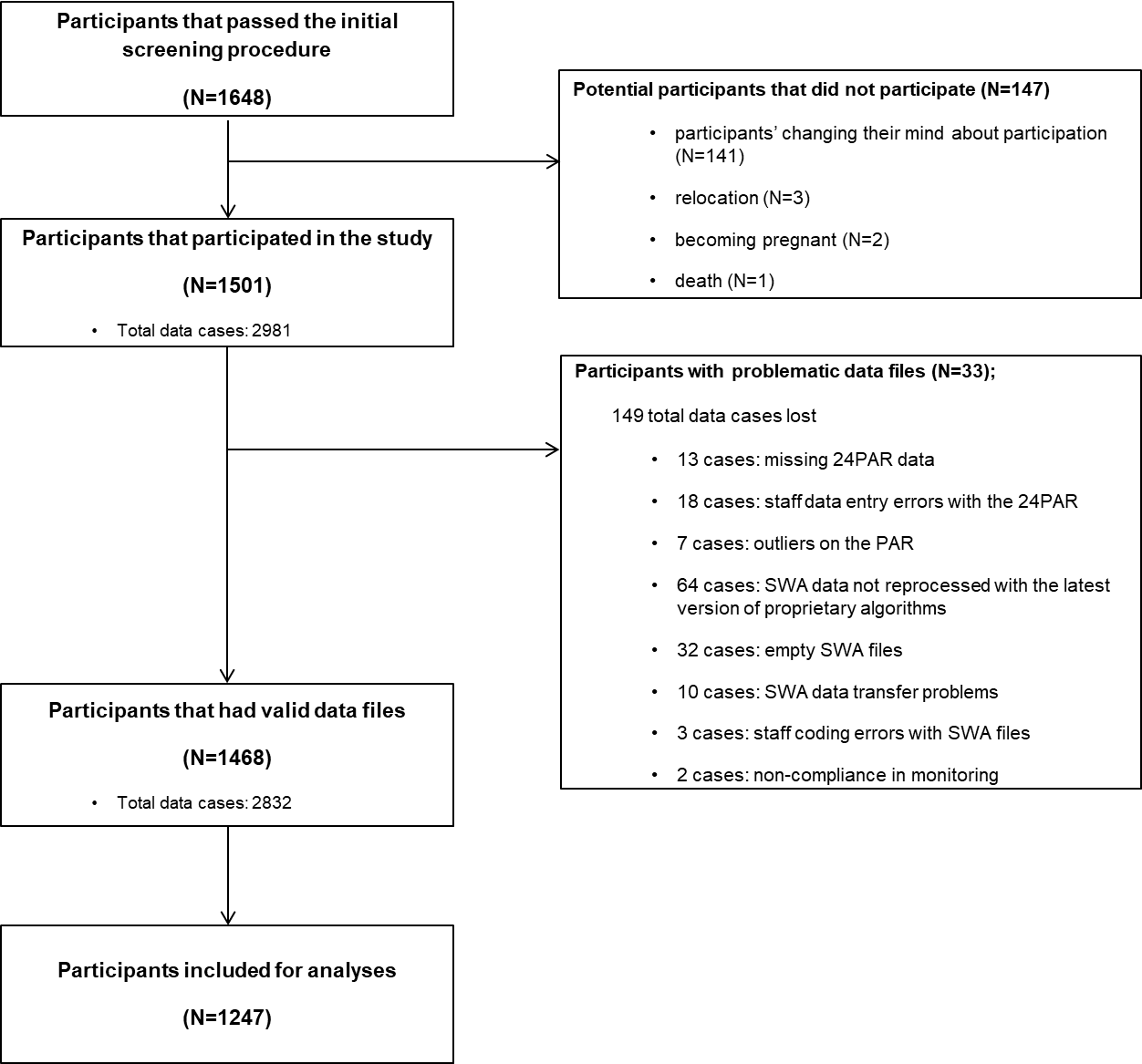


Supplementary Figure 1. A participant flow chart.


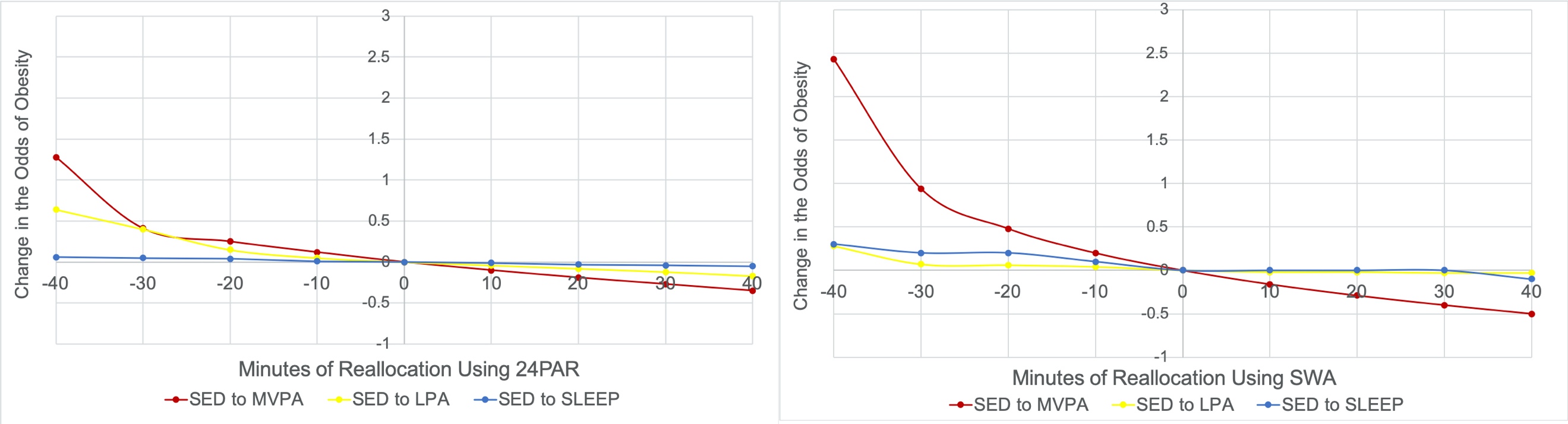


Supplementary Figure 2. Change in the odds of obesity for reallocation of time-use behaviour using 24-hour Physical Activity Recall and SenseWear Armband. Note: 24PAR stands for 24-hour Physical Activity Recall; SWA stands for SenseWear Armband; SLEEP is time sleeping; SED stands for Sedentary Time; LPA stands for Light Physical Activity; MVPA stands for Moderate-to-Vigorous Physical Activity.
